# *Prevotella loescheii* attenuates pregnancy‐induced hypertension by reshaping placental immunity and halting macrophage pyroptosis

**DOI:** 10.1002/ccs3.70099

**Published:** 2026-08-17

**Authors:** Jingjing Wu, Linlin Hu, Peipei Chen, Lihong Cai

**Affiliations:** ^1^ Obstetrics Department The Second Affiliated Hospital Fujian Medical University Fujian Quanzhou China

**Keywords:** placental microenvironment, pregnancy‐induced hypertension, *Prevotella loescheii*, pyroptosis, single‐cell RNA sequencing

## Abstract

Pregnancy‐induced hypertension (PIH) severely impairs placental function, yet its microenvironmental alterations and potential interventions remain unclear. Here, we combined single‐cell RNA sequencing (scRNA‐seq) with an in vivo model to investigate the protective effects of *Prevotella loescheii* (PL) in PIH. scRNA‐seq revealed that PIH reshaped placental cellular composition, reducing trophoblasts and myeloid cells while increasing smooth muscle cells, and disrupted cell–cell communication. Functional analyses identified oxidative stress (OS) and macrophage‐driven pyroptosis via the NLRP3 inflammasome as key pathological features. Trajectory analysis further demonstrated the depletion of Hofbauer cells, which was restored by PL. In vivo, PL significantly alleviated PIH phenotypes, including proteinuria and placental structural damage. Mechanistically, PL reduced proinflammatory cytokines, suppressed mitochondrial OS and pyroptosis, and partially restored short‐chain fatty acid metabolism. Collectively, these findings indicate that PL ameliorates PIH by modulating the placental immune microenvironment and metabolic homeostasis, providing a potential therapeutic strategy.

## Graphical abstract

*Prevotella loescheii* alleviates pregnancy‐induced hypertension (PIH) by reshaping the placental immune microenvironment, suppressing oxidative stress (OS)‐mediated NLRP3 pyroptosis, and restoring short‐chain fatty acid (SCFA) metabolic homeostasis. *P. loescheii* alleviates PIH by reshaping the placental immune microenvironment, suppressing OS‐mediated NLRP3 pyroptosis, and restoring SCFA metabolic homeostasis.

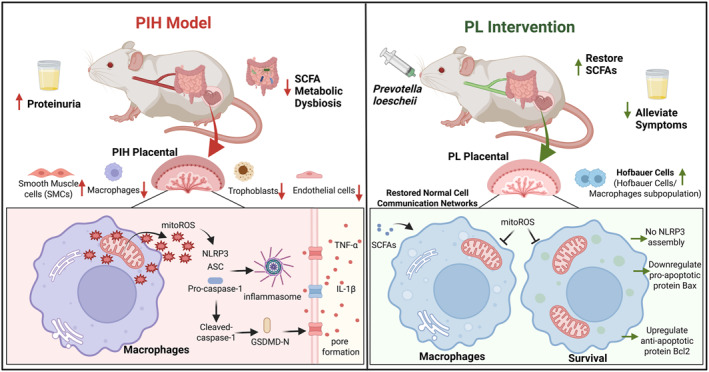

## 1 INTRODUCTION

Pregnancy‐induced hypertension (PIH) encompasses a spectrum of hypertensive disorders occurring during pregnancy, childbirth, or the puerperium, including gestational hypertension, preeclampsia‐eclampsia, and chronic hypertension with superimposed preeclampsia.^1^ Characterized primarily by systemic arteriolar spasm and multiple organ hypoperfusion, PIH manifests clinically as hypertension, edema, and proteinuria. It can rapidly progress to severe complications, such as placental abruption, disseminated intravascular coagulation, convulsions, and cardiorenal failure, making it a leading cause of maternal and perinatal morbidity and mortality globally.^2^, ^3^ Despite its high and rising incidence, current pharmacological interventions, mainly comprising antispasmodics, antihypertensives, and diuretics, offer limited symptomatic relief. Their clinical application is severely constrained by dosage limitations, adverse side effects, and strict safety requirements concerning maternal cardiovascular health and fetal development.^3^ Therefore, elucidating the underlying pathogenesis of PIH and developing novel, safe, and effective targeted therapies remain an urgent clinical imperative.

Accumulating evidence highlights the critical role of gut microbiota homeostasis in blood pressure regulation,^4^ with maternal gut dysbiosis emerging as a significant contributor to the pathogenesis of PIH.^5^ Previous studies have demonstrated a marked alteration in the gut microbiome of patients with PIH, characterized by a depletion of beneficial commensals (e.g., *Prevotella*, *Lachnospira*, and *Barnesiella*) and an overgrowth of opportunistic pathogens (e.g., *Enterobacter* and *Eggerthella*). This suggests that microbial perturbations and subsequent bacterial translocation are deeply implicated in PIH progression. Notably, *Prevotella loescheii* (PL), a specific species within the *Prevotella* genus, has recently shown potential protective effects against cardiovascular inflammation and metabolic disorders. For instance, recolonization with PL has been reported to inhibit the M1 polarization of colonic macrophages, reduce the production of interleukin‐1β (IL‐1β) and tumor necrosis factor (TNF)‐α, and mitigate immune‐related cardiotoxicity.^6^ Furthermore, its increased abundance correlates with improved glycemic control and attenuated oxidative stress (OS) in diabetic models.^7^ However, whether PL is involved in the pathological regulation of PIH and possesses therapeutic potential remains completely unexplored.

Short‐chain fatty acids (SCFAs)—primarily acetate, propionate, and butyrate—are vital microbial fermentation metabolites that regulate energy metabolism, immune homeostasis, and intestinal barrier integrity.^8^ Significant metabolic shifts in SCFAs have been identified in pregnancy‐related diseases, suggesting their role as potential biomarkers for obstetric complications.^9^ Crucially, butyrate supplementation has been shown to ameliorate hypertension and OS in preeclamptic rat models,^10^ whereas maternal SCFA supplementation can prevent the onset of gestational hypertension.^11^ SCFAs also promote trophoblast invasion and improve endothelial function.^12^, ^13^ Importantly, butyrate has been identified as a key functional metabolite of PL, largely mediating its anti‐inflammatory and antioxidant properties.^6^, ^7^

Mechanistically, SCFAs have been shown to modulate inflammatory responses in cardiovascular and metabolic diseases by targeting the NLRP3 inflammasome.^14^, ^15^ The NLRP3 inflammasome, comprising NLRP3, apoptosis‐associated speck‐like protein containing a CARD (ASC), and pro‐caspase‐1, promotes the maturation and release of proinflammatory cytokines (IL‐1β and IL‐18) and induces gasdermin D (GSDMD)‐mediated pyroptosis upon activation.^16^, ^17^, ^18^ In the context of PIH, NLRP3‐driven placental inflammation and pyroptosis are closely associated with impaired trophoblast invasion, endothelial injury, and subsequent blood pressure elevation.^19^, ^20^ This raises the intriguing hypothesis that the SCFA‐NLRP3 axis might serve as the pivotal signaling pathway through which PL exerts its protective effects against PIH.

To date, the specific roles of PL, its derived SCFAs, and their collective impact on placental inflammation in PIH have not been systematically investigated. In this study, we aimed to explore the gut microbiota‐metabolite‐placental inflammation axis. Utilizing an established PIH rat model combined with single‐cell transcriptomics, we comprehensively investigated the mechanistic role of PL and its SCFA metabolites in modulating the NLRP3‐pyroptosis pathway during PIH development. Our findings aim to provide novel mechanistic insights and highlight the potential of microbiome‐targeted microecological interventions for the prevention and management of PIH.

## 2 MATERIALS AND METHODS

### 2.1 Animals and experimental design

Healthy, specific pathogen‐free (SPF) pregnant Sprague–Dawley (SD) rats (gestational days [GD] 5–6, confirmed by the presence of a vaginal plug) were purchased from Beijing Vital River Laboratory Animal Technology Co., Ltd. All animals were housed in a temperature‐ (22 ± 2°C) and humidity‐controlled (50% ± 10%) SPF facility under a 12‐h light/dark cycle with ad libitum access to standard laboratory chow and sterile water. On GD 12, pregnant rats were randomly allocated into three groups (*n* = 6 per group): control, PIH model (model), and PL intervention. To establish the PIH model, rats in the model and PL groups received daily intraperitoneal (i.p.) injections of NG‐nitro‐L‐arginine methyl ester (L‐NAME; 50 mg/kg/d; Yuanye Bio‐Technology) from GD 12 to GD 18, whereas the control group received equal volumes of sterile saline. For microbial intervention, the standard PL strain (DSM 19665) was acquired from the German Collection of Microorganisms and Cell Cultures (DSMZ). After resuscitation and cultivation, the bacterial suspension was centrifuged and resuspended in sterile anaerobic phosphate‐buffered saline (PBS). During the modeling period (GD 12–18), pregnant rats in the PL group received daily intragastric (i.g.) administration of the bacterial suspension (totaling 10^10^ colony‐forming unit per rat), whereas the control and model groups received equal volumes of sterile PBS. On GD 21, after final blood pressure measurements and urine collection, the rats were euthanized via CO_2_ asphyxiation. Placental tissues, blood, and fecal samples were rapidly harvested for subsequent analyses. All animal experimental protocols were approved by the Experimental Animal Ethics Committee of Fujian Medical University (Approval No.: IACUC FJMU 2023‐Y‐1224) and were conducted in accordance with institutional and national guidelines for animal welfare.

### 2.2 Blood pressure and 24‐h urine protein measurement

Systolic blood pressure (SBP) was monitored via the noninvasive tail‐cuff method using a Medlab blood pressure analysis system (Medlab, Calvin) on GD 12 (baseline), 15, 19, and 21. To minimize stress, rats were acclimatized to the restraining holder and prewarmed for several days prior to actual recordings. At least 10 consecutive stable readings were acquired per rat, and the average was recorded. The PIH model was considered successfully established if the SBP on GD 15 increased by ≥20 mm Hg compared to the GD 12 baseline. Concurrently, pregnant rats were placed individually in metabolic cages with free access to water to collect 24‐h urine. The urine volume was recorded, and after centrifugation, the total 24‐h urine protein concentration in the supernatant was quantified using a rat urine protein enzyme‐linked immunosorbent assay (ELISA) kit/bicinchoninic acid assay.

### 2.3 Preparation of placental single‐cell suspensions

Fresh placental tissues from each group were collected, minced, and dissociated using TrypLE Express Enzyme (Thermo Fisher Scientific) at 37°C for 10 min. Subsequently, a complex collagenase cocktail (Gibco) was added and incubated at 37°C for another 10 min. The digested suspension was passed through a 40‐μm cell strainer to remove debris, and erythrocytes were eliminated using an red blood cell lysis buffer (Solarbio) for 90 s. To minimize individual variation, single‐cell suspensions from rats within the same group were pooled for downstream single‐cell RNA sequencing (scRNA‐seq) analysis.

### 2.4 scRNA‐seq library construction and preprocessing

Cell capture and 3′ transcriptomic library construction were performed using the DNBelab C‐TaiM 4 platform (MGI Tech Co., Ltd.). Two types of sequencing libraries were generated: cDNA libraries (yielding ∼550 million reads) and Qlig9 libraries (yielding ∼50 million reads). Sequencing was executed on the DNBSEQ‐T7 platform, and raw FASTQ sequence file format data were generated. Upstream data processing was conducted using DNBc4tools v2.1.2. Reads were mapped to the *Rattus norvegicus* reference genome (Rnor 6.0), followed by barcode correction, unique molecular identifier deduplication, and count generation to construct the gene‐by‐cell expression matrix. Ambient RNA contamination and low‐quality/multiplet artifacts were assessed and filtered prior to downstream analysis.

### 2.5 scRNA‐seq data analysis

The R package Seurat (v5.0.1) was utilized for quality control and downstream analysis. Cells expressing fewer than 200 genes, genes expressed in fewer than 3 cells, and cells with >10% mitochondrial gene expression were excluded. Batch effects across groups were corrected using the Harmony package. Dimensionality reduction and clustering were performed using Uniform Manifold Approximation and Projection (resolution = 0.5). Cell types were manually annotated based on canonical markers, followed by the generation of cell atlases, marker bubble plots, and cell proportion bar charts. Differentially expressed genes (DEGs) were identified using the FindMarkers function. Gene Ontology and Kyoto Encyclopedia of Genes and Genomes (KEGG) pathway enrichment analyses were performed to determine biological functions. Critical cell subpopulations were selected based on AUCell (v1.22.0) scoring and their correlation with pyroptosis/OS‐related gene (PDRG/OSRG, Supplementary Tables S1 and S2) signatures.

### 2.6 Cell–cell communication analysis

Intercellular communication networks were inferred using the CellChat algorithm (v1.6.1) to identify key receptor–ligand interactions and signaling pathways. This analysis was conducted both within subsets of critical cell populations and between these key clusters and other placental cells, elucidating the coordinated cellular interactions driving PIH pathogenesis.

### 2.7 Pseudotime trajectory analysis

Single‐cell trajectory analysis was performed using the R package Monocle 2 to reconstruct the developmental lineages and cellular states associated with PIH. The dynamic expression patterns of key genes along the pseudotime trajectory were mapped to determine their potential roles in disease progression.

### 2.8 Gene regulatory network (pySCENIC) analysis

Transcription factor (TF) regulatory networks within each cell type were inferred using the Python implementation of SCENIC (pySCENIC). Coexpression modules were calculated via GRNBoost, and TF binding motifs were validated using RcisTarget. TF activity at the single‐cell level was quantified via AUCell and visualized using heatmaps generated by the pheatmap package.

### 2.9 Quantification of SCFAs

The concentrations of seven SCFAs in the samples were quantified using gas chromatography‐mass spectrometry. Briefly, 25 mg of the sample was homogenized in 1 mL of ultrapure water using a bead mill (40 Hz, 4 min) and ultrasonicated in an ice‐water bath for 5 min (repeated 3 times). After centrifugation (5000 × g, 20 min, 4°C), 0.8 mL of the aqueous supernatant was acidified with 0.1 mL of 50% H_2_SO_4_ and extracted with 0.8 mL of methyl tert‐butyl ether containing 2‐ethylbutyric acid (200 μg/mL) as an internal standard. The organic layer was collected and analyzed using an Agilent 7890A/5975C system (Agilent Technologies) equipped with a polyethylene glycol‐based capillary column (30 m × 0.25 mm, 0.5 μm). The absolute concentrations of SCFAs (μg/g) were calculated based on internal standard‐corrected calibration curves (ANPEL).

### 2.10 Histological evaluation and TUNEL assay

Placental tissues were fixed, dehydrated, paraffin embedded, and sectioned (4–5 μm). Sections were deparaffinized, rehydrated, and stained with hematoxylin and eosin for histopathological observation under a light microscope.

For apoptosis assessment, sections were assayed using a commercial terminal deoxynucleotidyl transferase‐mediated dUTP nick‐end labeling (TUNEL) Apoptosis Detection Kit (Yeasen Biotechnology) according to the manufacturer’s protocol. Briefly, sections were blocked with 3% bovine serum albumin (BSA) (Sigma‐Aldrich) at 37°C for 15 min, equilibrated for 10 min, and incubated with the TdT enzyme reaction mixture at 37°C for 60 min. After PBS washing, apoptotic cells were visualized and quantified using an inverted fluorescence microscope (IX73, Olympus).

### 2.11 Measurement of ROS

Fresh placental samples were embedded in optimal cutting temperature compound compound (Sakura Finetek) and cryosectioned using a cryostat (CM3050S, Leica). Intracellular reactive oxygen species (ROS) levels in the tissues were detected using a 2′,7′‐dichlorodihydrofluorescein diacetate fluorescent probe (Beyotime), following the manufacturer’s instructions. Images were captured under consistent parameters using the Olympus IX73 fluorescence microscope.

### 2.12 Enzyme‐Linked Immunosorbent Assay

Peripheral blood was collected, and serum was isolated via centrifugation. The levels of inflammatory cytokines and OS markers, including IL‐1β, TNF‐α, IL‐10, TGF‐β, malondialdehyde (MDA), and superoxide dismutase (SOD), were determined using commercial ELISA/colorimetric assay kits (Meimian Industrial Co., Ltd.) in accordance with the manufacturer’s guidelines. The optical density of each well was measured at 450 nm using a microplate reader.

### 2.13 Western blotting

Placental tissues were homogenized in radioimmunoprecipitation assay buffer lysis buffer supplemented with protease inhibitors (Beyotime). Protein concentrations were determined using a Nano300 spectrophotometer. Equal amounts of protein were separated by sodium dodecyl sulfate–polyacrylamide gel electrophoresis and transferred onto polyvinylidene fluoride membranes (Millipore). After blocking with 5% nonfat milk, membranes were incubated overnight at 4°C with primary antibodies: MMP9 (AF5228, Affinity Biosciences), MMP2 (AF5330, Affinity), ASC (bsm‐61273R, Bioss), cleaved‐caspase‐1 (89332S, Cell Signaling Technology), GSDMD (20770‐1‐AP, Proteintech), NLRP3 (30109‐1‐AP, Proteintech), pro‐caspase‐1 (ab179515, Abcam), Bcl‐2 (A19693, ABclonal), Bax (50599‐2‐Ig, Proteintech), caspase‐3 (9662S, Cell Signaling Technology), cleaved‐caspase‐3 (9661S, Cell Signaling Technology), and glyceraldehyde‐3‐phosphate dehydrogenase (10494‐1‐AP, Proteintech). Membranes were then incubated with HRP‐conjugated secondary antibodies (Abbkine) and visualized using an enhanced chemiluminescence chemiluminescence kit (Tanon Science & Technology).

### 2.14 Immunofluorescence staining

To evaluate macrophage polarization, tissue sections were permeabilized, blocked with 5% BSA, and incubated overnight with specific primary antibodies against CD86 (M1 marker; SC‐19617, Santa Cruz Biotechnology), CD206 (M2 marker; SC‐376108), and CD68 (pan‐macrophage marker; 28058‐1‐AP, Proteintech). Sections were subsequently incubated with fluorochrome‐conjugated secondary antibodies (CST). Nuclei were counterstained with 4′,6‐diamidino‐2‐phenylindole (Beyotime). Images were captured using a fluorescence microscope.

### 2.15 Statistical analysis

All bioinformatics analyses of scRNA‐seq data were conducted using R software (version 4.3.3; R Foundation) with specific packages including Seurat, CellChat, and clusterProfiler. Other experimental data were analyzed using GraphPad Prism (version 9.0; GraphPad Software). Data are expressed as the mean ± standard deviation (SD). For comparisons among three groups, statistical significance was determined using one‐way analysis of variance followed by Tukey’s post‐hoc test (for normally distributed data) or the Kruskal–Wallis nonparametric test. A value of *p* < 0.05 was considered statistically significant.

## 3 RESULTS

### 3.1 Single‐cell transcriptomics delineates alterations in placental cell composition and remodeling of cell communication networks in PIH

**FIGURE 1:**
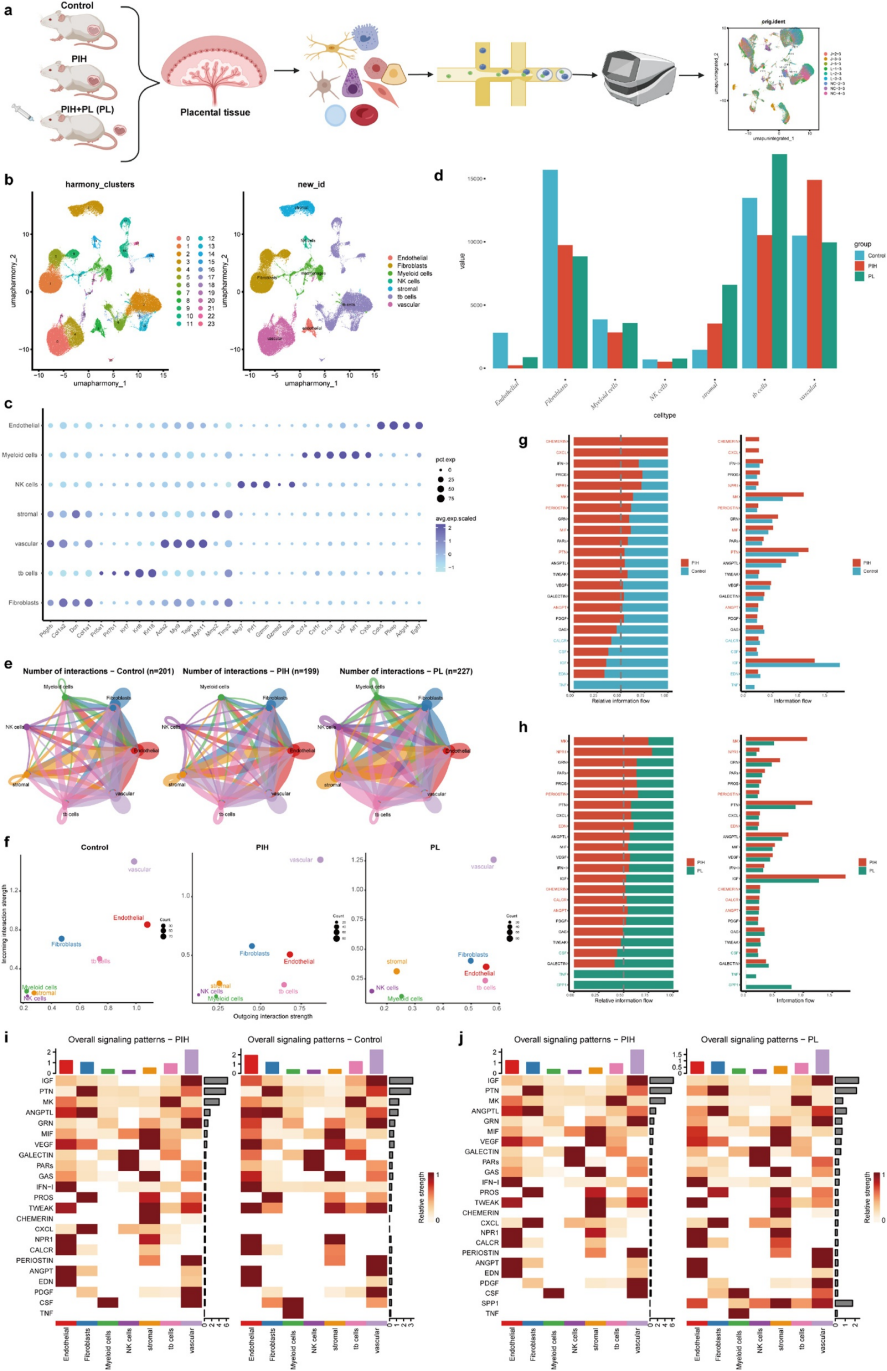
Single‐cell transcriptomic atlas of rat placental tissues. (A) Schematic workflow of the single‐cell RNA sequencing experiment for placental tissues from the three groups. (B) Uniform Manifold Approximation and Projection plot visualizing the major cell types in the placenta. (C) Bubble plot illustrating the expression of canonical marker genes across different cell types. (D) Stacked bar charts showing the relative proportions of each cell type across the control, model, and *Prevotella loescheii* (PL) intervention groups. (E) Circle plots showing the inferred intercellular communication networks for the control, PIH, and PL groups. The total numbers of interactions in each group are 201 (control), 199 (PIH), and 227 (PL), respectively. (F) Bubble plot comparing the signaling pathway strengths across different groups. (G, H) Bar charts demonstrating the relative contribution and absolute strength of specific signaling pathways. (I, J) Heatmaps depicting the relative interaction strength of signaling networks among the identified cell types in different groups. PIH, pregnancy‐induced hypertension.

To systematically elucidate the impact of PIH on the placental microenvironment, we first performed scRNA‐seq on placental tissues from the normal control group, PIH model group, and PL intervention group (Figure 1A). After data integration and unsupervised clustering, a total of 23 cell clusters were identified. Based on the expression of canonical marker genes, these clusters were annotated into seven major cell types: trophoblasts, endothelial cells, myeloid cells, natural killer (NK)/T cells, stromal cells, smooth muscle cells (SMCs), and fibroblasts (Figure 1B,C). Cell proportion analysis revealed that PIH significantly altered the cellular landscape of the placenta. The proportions of myeloid cells, trophoblasts, NK cells, and endothelial cells were decreased in the PIH model group, whereas the SMC population was increased (Figure 1D). Notably, these cellular abnormalities were restored to varying degrees in the PL intervention group.

Cell–cell communication network analysis suggested that PIH led to a significant restructuring of the interaction network within placental tissues (Figure 1E). Compared to the control group, the interaction strength between SMCs and endothelial cells was markedly enhanced in the PIH model group, whereas the communication between trophoblasts and SMCs was inhibited. Crucially, PL intervention partially reversed this aberrant trend (Figure 1F). At the signaling pathway level, inflammation‐related pathways such as angiopoietin, NRP1, and midkine were significantly enriched in PIH, whereas TNF and colony‐stimulating factor (CSF) signaling were primarily enriched in the control and intervention groups (Figure 1G,H). Heatmap clustering further confirmed that PIH promoted the enhancement of inflammatory signals, including PARs and CD40, while concurrently inhibiting TNF signaling in myeloid cells and CSF signaling in SMCs. These results collectively indicate that PIH disrupts the intrinsic immune regulatory network of the placenta, whereas PL intervention helps restore homeostatic cell–cell communication (Figure 1I,J).

### 3.2 Differential expression and functional enrichment analysis identify oxidative stress and pyroptosis as key pathological events in PIH

**FIGURE 2:**
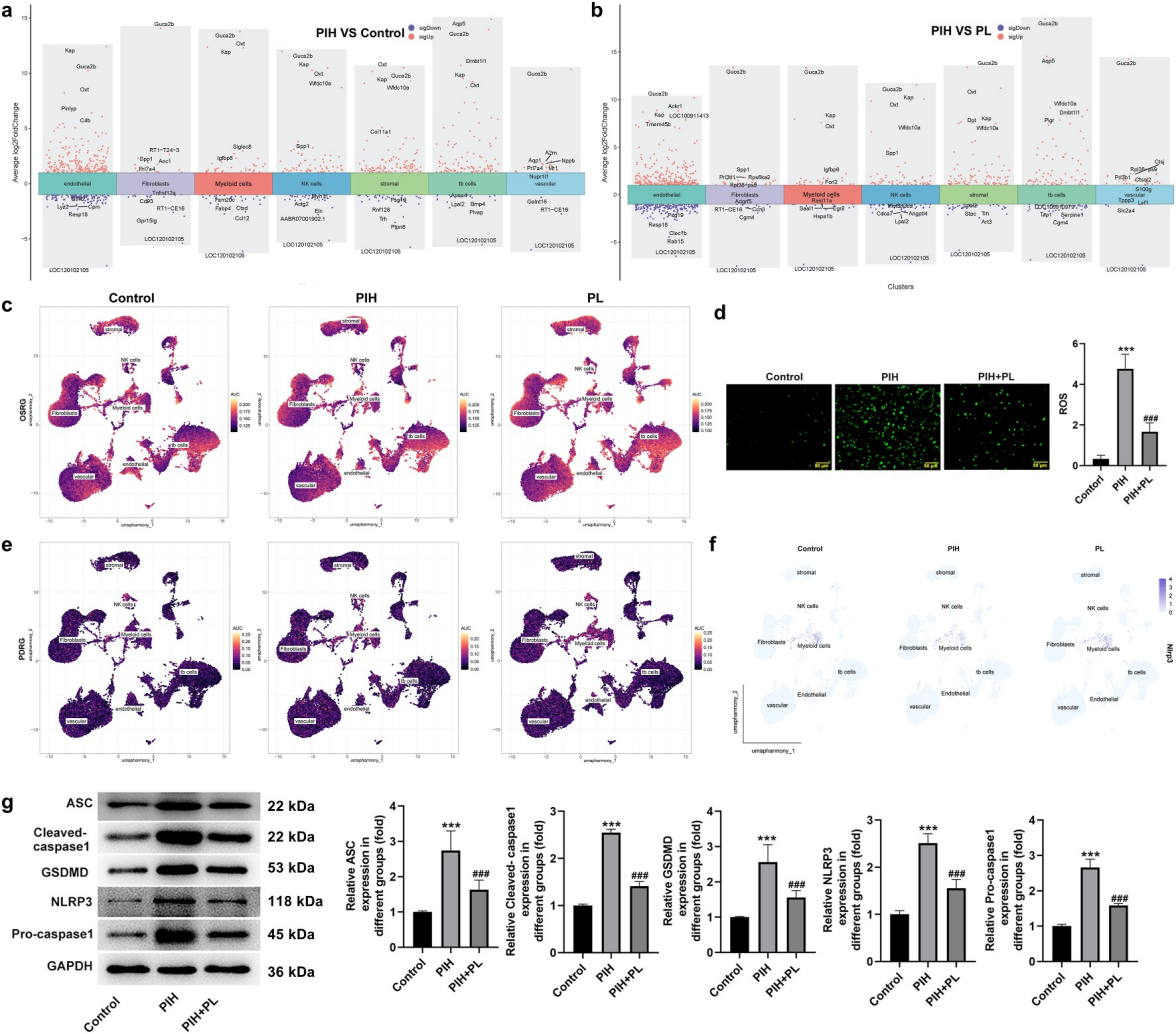
Identification of differentially expressed genes (DEGs) and profiling of oxidative stress (OS) and pyroptosis signatures in pregnancy‐induced hypertension placentas. (A, B) Volcano plots showing the DEGs across various cell types for the model versus control group (left) and the model versus *Prevotella loescheii* group (right). (C) Violin plots displaying the AUCell scores for the OS‐related gene set across cell types. (D) Representative images and quantification of reactive oxygen species fluorescence staining in placental tissues. (E) Violin plots depicting the AUCell scores for the pyroptosis‐related gene set. (F) FeaturePlot showing the specific expression distribution of NLRP3 across groups. (G) Relative protein expression levels of NLRP3, ASC, pro‐caspase‐1, cleaved‐caspase‐1, and gasdermin D in placental tissues, as determined by Western blotting. Data are presented as the mean ± standard deviation (*n* = 3 per group for Western blot). *p < 0.05, **p < 0.01, ***p < 0.001 versus Control; ^#^ *p* < 0.05, ^##^ *p* < 0.01, ^###^ *p* < 0.001 versus PIH. ASC, apoptosis‐associated speck‐like protein containing a CARD; PIH, pregnancy‐induced hypertension.

Based on the scRNA‐seq data, we analyzed DEGs among the major cell types across the different treatment groups. Using standard screening criteria (|log2FC| > 1 and *p* < 0.05), distinct cell types exhibited heterogeneous transcriptional responses to PIH (Figure 2A,B). Notably, endothelial cells presented the highest number of DEGs, suggesting significant transcriptional remodeling in this population during PIH pathogenesis.

To further investigate the role of OS and cell death pathways, we performed AUCell scoring on each cell type using predefined gene sets for OS and pyroptosis (Figure 2C,E). Compared with different groups, there are significant differences in the OS and pyroptosis scores of endothelial cells, myeloid cells, and SMCs, indicating cell‐type‐specific responses to stress and death signals under PIH conditions. Importantly, these effects were reversed in the PL group. Given the central role of myeloid cells in inflammatory regulation, we performed KEGG pathway enrichment analysis on their DEGs. The results showed that DEGs in myeloid cells were significantly enriched in multiple signaling pathways closely related to OS, inflammatory responses, and cell death, suggesting a pivotal role for myeloid cells in PIH pathology (Figure S1). To validate these findings, mitochondrial ROS (mitoROS) levels in placental tissues were assessed using a fluorescent probe (Figure 2D). Consistent with the transcriptional data, mitoROS levels were markedly increased in the PIH group. FeaturePlot analysis further confirmed that NLRP3 was highly specifically expressed in myeloid cells (Figure 2F). Correspondingly, protein expression analysis showed significant upregulation of NLRP3, ASC, pro‐caspase‐1, cleaved‐caspase‐1, and GSDMD in placental tissues of the PIH group (Figure 2G). In the PL intervention group, the expression levels of these pyroptosis‐related proteins were significantly reduced, indicating that PL treatment effectively mitigates OS and pyroptosis in the PIH placenta.

### 3.3 Myeloid subpopulation identification and pseudotime trajectory analysis reveal specific responses to PIH

**FIGURE 3:**
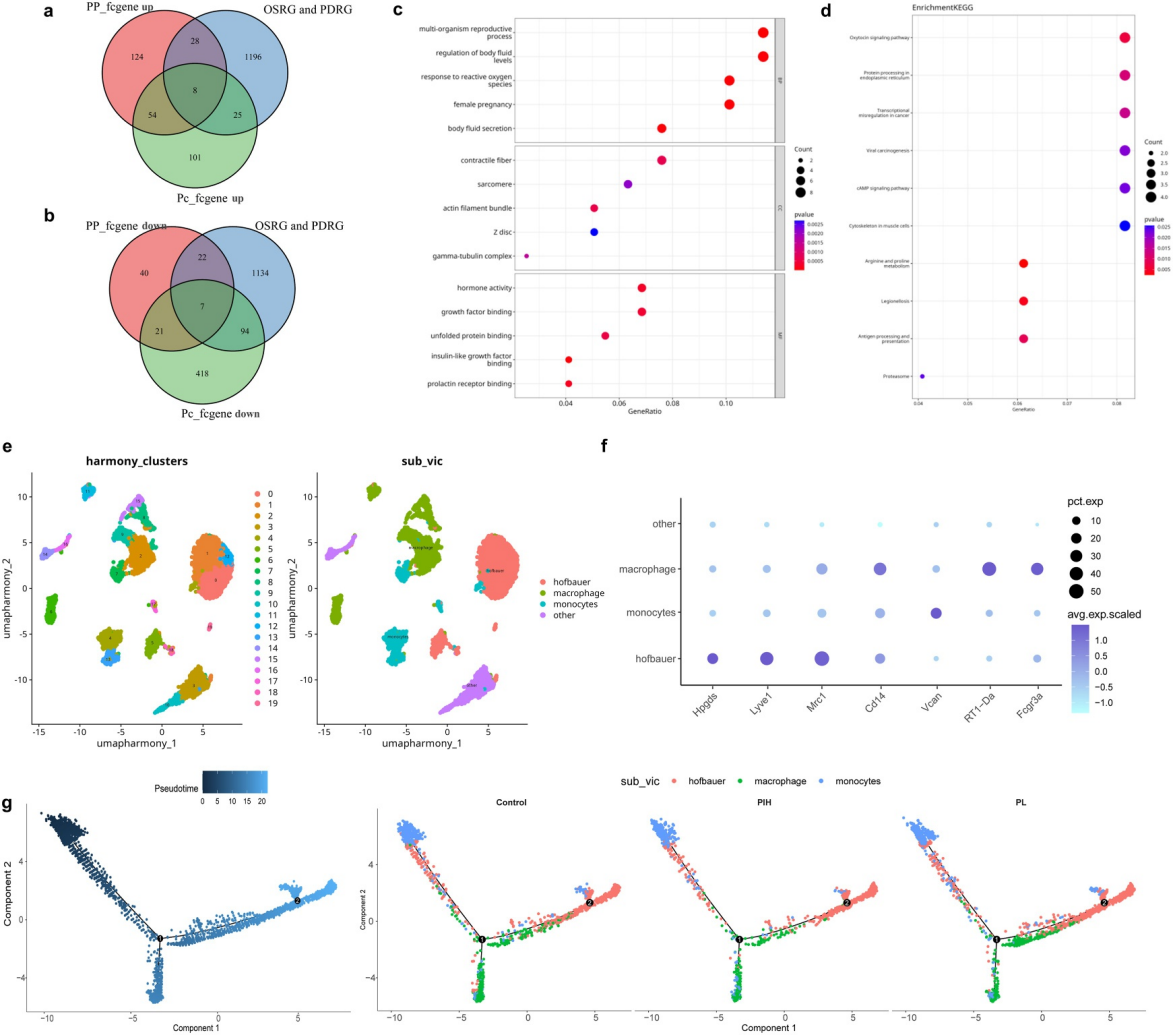
Subclustering and pseudotime trajectory analysis of placental myeloid cells. (A, B) Venn diagrams illustrating the intersection of DEGs with OS‐related genes and pyroptosis‐related genes. The upper panel (A) shows upregulated genes, whereas the lower panel (B) shows downregulated genes (Pc: PIH vs. Control; PP: PIH vs. PL). (C, D) Gene Ontology (C) and Kyoto Encyclopedia of Genes and Genomes pathway (D) enrichment analyses for the shared DEGs. (E) Uniform Manifold Approximation and Projection plot of the reclustered myeloid subpopulations. (F) Bubble plot showing the expression of specific marker genes across myeloid subclusters, highlighting the transcriptomic divergence between Hofbauer cells and general macrophages. (G) Pseudotime trajectory distribution of myeloid cells and their distribution density across the different treatment groups. DEG, differentially expressed gene; PIH, pregnancy‐induced hypertension; PL, *Prevotella loescheii*.

To further dissect the heterogeneity of myeloid cells under PIH conditions, placental myeloid cells were subjected to detailed subclustering. The intersection of macrophage DEGs with OSRG and PDRG sets yielded a total of 15 key overlapping genes (8 upregulated and 7 downregulated) (Figure 3A,B). Functional enrichment revealed that these genes were primarily involved in the cAMP and oxytocin signaling pathways (Figure 3C,D). Based on these transcriptional features, myeloid cells were reclustered into three main subpopulations: Hofbauer cells (HBCs), macrophages, and monocytes (Figure 3E). Although HBCs belong to the macrophage lineage, subclustering analysis revealed substantial transcriptomic divergence, justifying their designation as a distinct population. Specifically, HBCs were characterized by high expression of tissue‐resident and immunomodulatory markers (Hpgds, Lyve1, and Mrc1), suggesting a specialized role in maintaining maternal‐fetal tolerance. In contrast, the general macrophage cluster exhibited a distinct signature enriched in immune surveillance and antigen presentation, marked by high expression of RT1‐Da, Fcgr3a, and Cd14 (Figure 3F).

**FIGURE 4:**
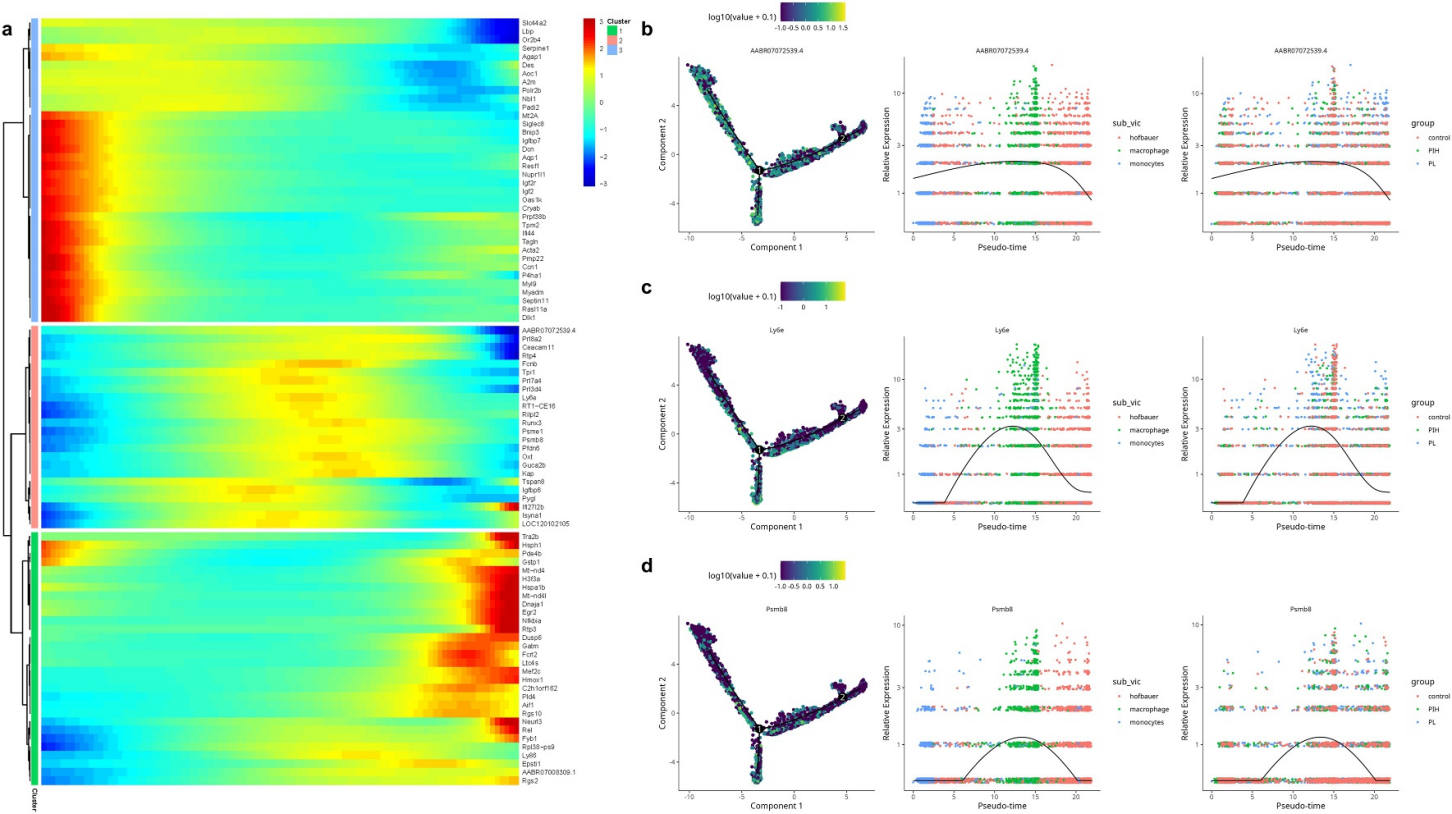
Dynamic expression of key genes along the pseudotime trajectory in myeloid subclusters. (A) Heatmap showing the expression dynamics of representative genes over pseudotime differentiation. (B–D) Pseudotime heatmaps demonstrating the expression trends of key genes associated with the probiotic intervention effect (AABR07072539.4, Ly6e, and Psmb8).

Pseudotime trajectory analysis revealed that macrophage differentiation was organized into two key branch nodes and three potential developmental branches. Although the overall shape of the differentiation trajectory was not drastically altered by the disease state, the proportion of HBCs was noticeably reduced in the PIH model group but recovered in the PL intervention group, establishing a clear link between PL treatment and HBC population dynamics (Figure 3G). Further investigation of gene expression dynamics along the pseudotime trajectory identified distinct distribution trends for key DEGs, such as Ly6e and Psmb8, during differentiation (Figure 4).

### 3.4 Myeloid cells’ TF‐target gene network analysis highlights the restorative effect of PL on PIH‐induced transcriptional dysregulation

**FIGURE 5:**
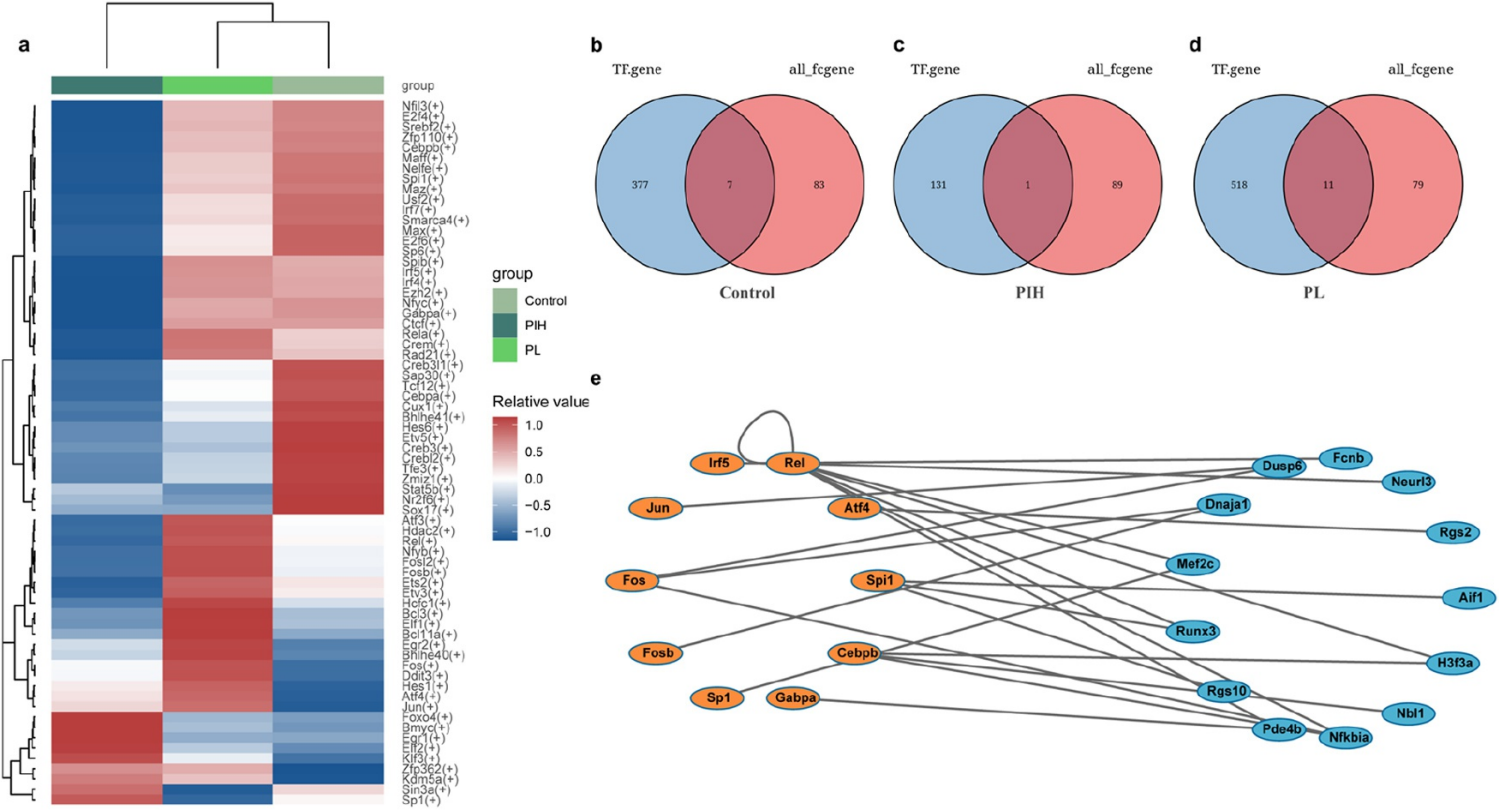
Transcription factor (TF) regulatory network analysis in myeloid cells. (A) Heatmap illustrating the TF activity patterns (AUC scores) across different groups, analyzed by Python implementation of SCENIC. (B–D) Venn diagrams showing the intersection between active TFs and the shared differentially expressed genes among groups. (E) The inferred interaction network between key TFs and their downstream target genes.

To evaluate the transcriptional regulatory status of myeloid cells, we performed TF activity analysis using pySCENIC. The global TF activity pattern in the PL group was highly similar to that of the control group, whereas the PIH group clustered distinctly apart (Figure 5A), indicating a significant disruption of the macrophage transcriptional regulatory network under PIH. By integrating TF activity with DEG data, an intersection analysis identified 14 key target genes, including Aif1, H3f3a, Nbl1, Nfkbia, Pde4b, Rgs10, Runx3, Mef2c, Dnaja1, Dusp6, Fcnb, Neurl3, Rel, and Rgs2 (Figure 5B–D, Supplementary Tables S3–S5). These genes exhibited significant differential expression across groups; notably, OSRGs such as Aif1 and Nfkbia were downregulated in the PIH group. Gene regulatory network analysis revealed that multiple TFs coregulated these 14 target genes to form specific regulatory modules. Importantly, in the PL intervention group, this network architecture and the corresponding gene expression patterns were markedly remodeled, reverting toward normal physiological levels (Figure 5E).

### 3.5 PL intervention ameliorates systemic injury, placental structural/functional impairment, and metabolic disorders in PIH rats

**FIGURE 6:**
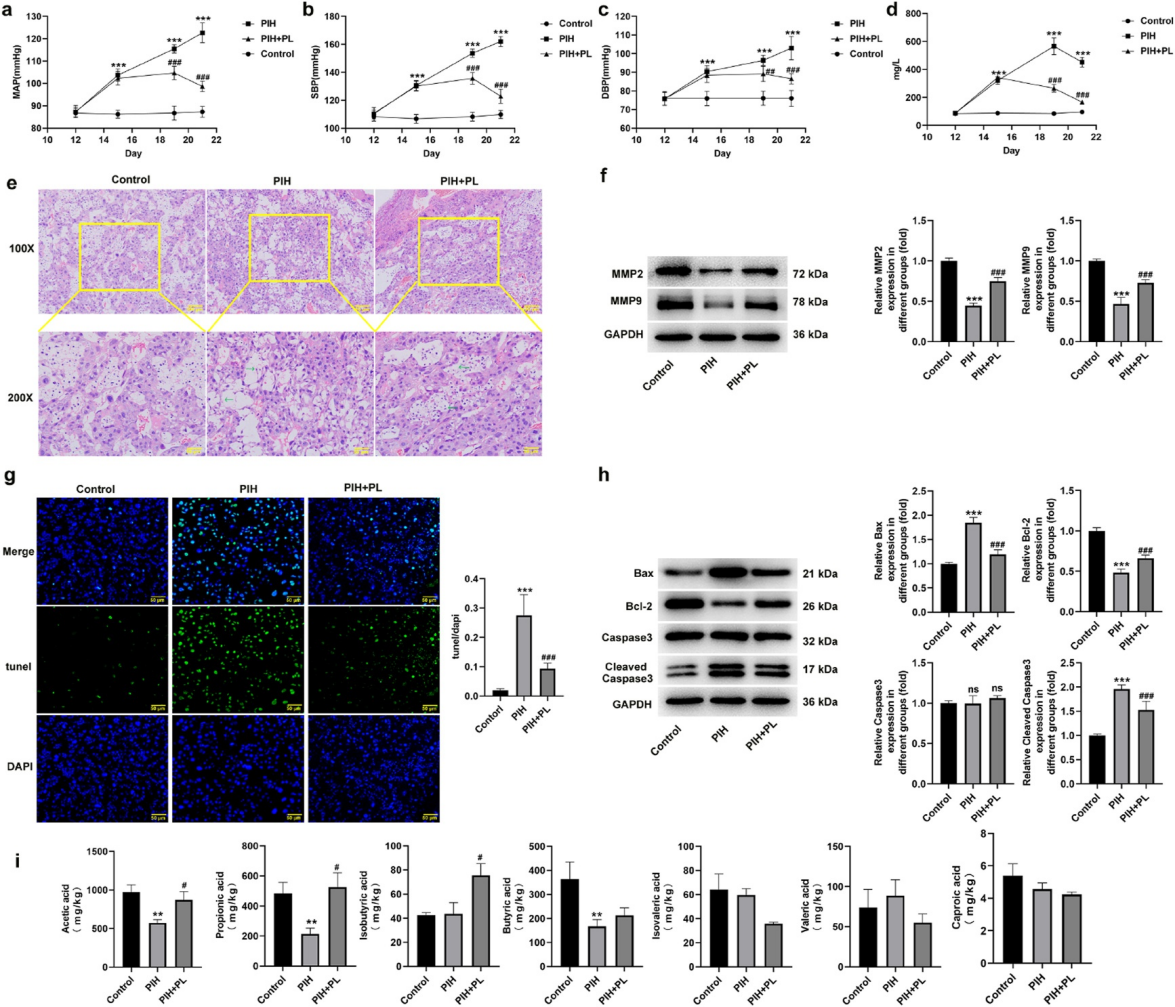
*Prevotella loescheii* intervention alleviates systemic symptoms, placental structural damage, and SCFA metabolic disorders in pregnancy‐induced hypertension rats. (A–D) Detection of systolic blood pressure and 24‐h urine protein levels at different gestational days. (E) Representative hematoxylin and eosin staining images of placental tissues from each group. (F) Relative protein expression levels of MMP2 and MMP9 in placental tissues, determined by Western blotting. (G) Representative images of TUNEL staining for detecting placental apoptosis. Scale bar = 50 μm. (H) Relative protein expression levels of Bax, Bcl‐2, caspase‐3, and cleaved‐caspase‐3 in placental tissues. (I) Targeted metabolomic quantification of short‐chain fatty acids in the samples. Data are presented as the mean ± standard deviation (*n* = 3 per group for Western blot; *n* = 6 for others). *p < 0.05, **p < 0.01, ***p < 0.001 versus Control; ^#^ *p* < 0.05, ^##^ *p* < 0.01, ^###^ *p* < 0.001 versus PIH. PIH, pregnancy‐induced hypertension; SCFA, short‐chain fatty acid; TUNEL, terminal deoxynucleotidyl transferase‐mediated dUTP nick‐end labeling.

To validate the transcriptomic findings in vivo, we evaluated the therapeutic efficacy of PL in the PIH rat model. Assessment of 24‐h urine protein levels on GD 12, 15, 19, and 21 revealed that proteinuria significantly increased in the PIH group starting from GD 15 and progressively worsened. Conversely, PL intervention significantly reduced urine protein levels on GD 19 and 21 (Figure 6A–D). Histological evaluation via H&E staining was performed to assess placental architecture (Figure 6E). Placentas from the control group exhibited normal, well‐organized villous morphology. In contrast, placentas from the PIH group demonstrated severe structural damage, characterized by disorganized labyrinthine architecture, prominent villous atrophy, and localized cellular damage/necrosis (green arrows). After PL intervention, these pathological alterations were substantially ameliorated, demonstrating a marked restoration of structural integrity.

Because placental structural remodeling is closely tied to trophoblast invasive capacity, we evaluated the expression of matrix metalloproteinases (MMP2 and MMP9) (Figure 6F). Compared to the control group, MMP2 and MMP9 expression was significantly reduced in PIH placentas but was prominently upregulated by PL intervention, indicating a restoration of trophoblast invasive function. Furthermore, placental cell death was assessed via TUNEL staining. The proportion of TUNEL‐positive apoptotic cells was markedly increased in the PIH group but significantly attenuated by PL treatment (Figure 6G). Western blot analysis of canonical apoptosis‐related proteins corroborated these findings: Pro‐apoptotic Bax and cleaved‐caspase‐3 were upregulated, whereas antiapoptotic Bcl‐2 was downregulated in PIH placentas. PL intervention effectively reversed these trends, confirming the inhibition of placental apoptosis (Figure 6H). Additionally, profiling of SCFAs revealed significant variations in five of the seven tested SCFAs among the groups, excluding valeric and caproic acids (Figure 6I). The PIH group exhibited pronounced SCFA metabolic dysbiosis, which was partially corrected by PL administration. This suggests that PIH is accompanied by an imbalance in gut‐derived metabolites and that PL may exert its protective effects by modulating these SCFA metabolic pathways.

### 3.6 PL mitigates placental inflammation, oxidative stress, and mitochondrial dysfunction in PIH rats

**FIGURE 7:**
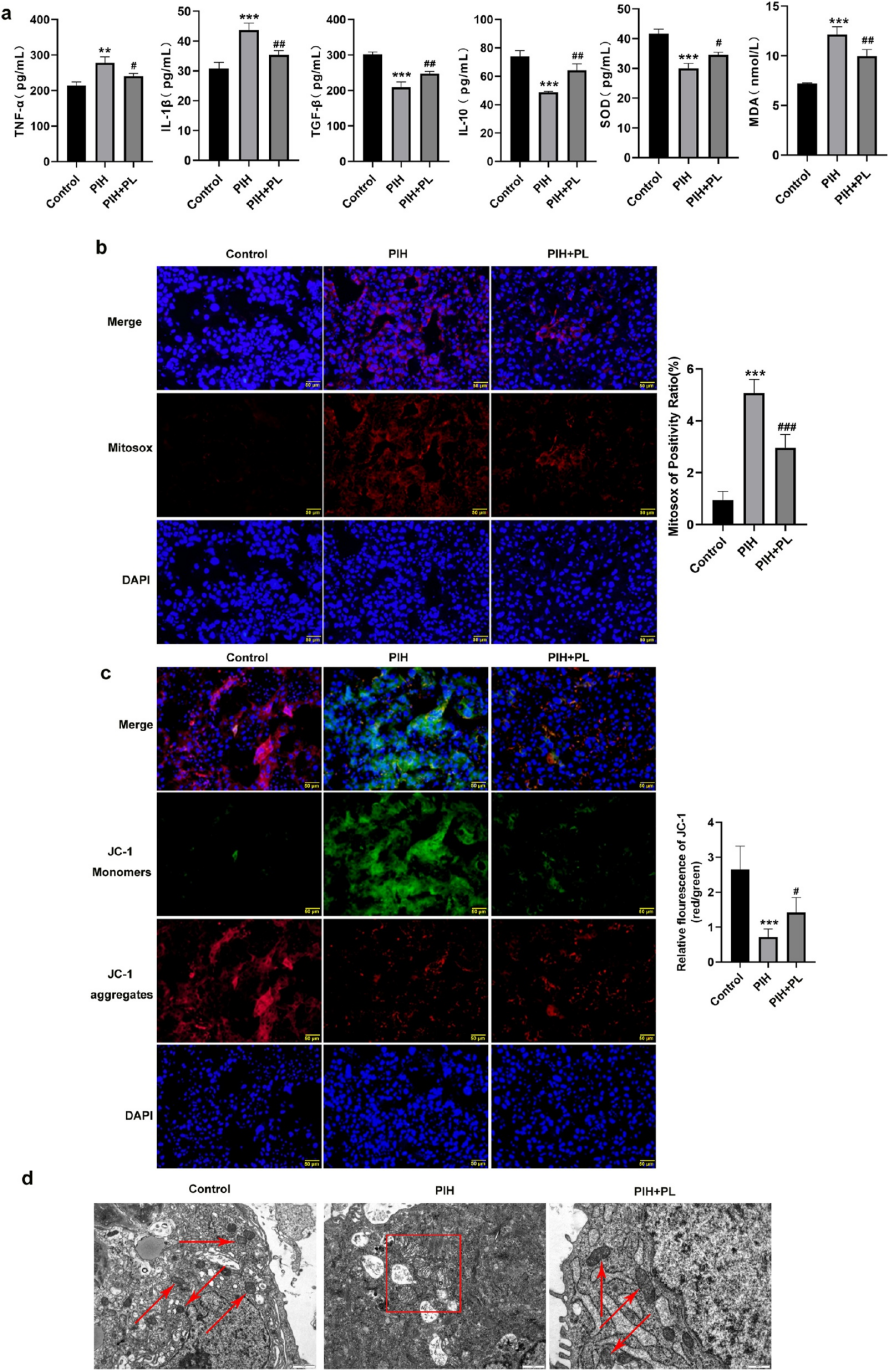
*Prevotella loescheii* intervention mitigates peripheral inflammation, placental OS, and mitochondrial dysfunction in pregnancy‐induced hypertension rats. (A) Enzyme‐linked immunosorbent assay quantification of inflammatory cytokines and OS markers in peripheral blood serum samples. (B) Representative fluorescence images of mitochondrial reactive oxygen species staining detecting mitochondrial superoxide in placental tissues. Scale bar = 50 μm. (C) JC‐1 staining for the assessment of mitochondrial membrane potential in placental tissues. Scale bar = 50 μm. (D) Representative transmission electron microscopy images revealing the ultrastructure of placental tissues across the three groups. Data are presented as the mean ± standard deviation (*n* = 3 per group). *p < 0.05, **p < 0.01, ***p < 0.001 versus Control; ^#^ *p* < 0.05, ^##^ *p* < 0.01, ^###^ *p* < 0.001 versus PIH. OS, oxidative stress; PIH, pregnancy‐induced hypertension.

To comprehensively evaluate inflammation and OS, ELISA was performed on placental tissues. Compared to the control group, the levels of proinflammatory cytokines (TNF‐α and IL‐1β) and lipid peroxidation (MDA) were significantly elevated in the PIH group, whereas anti‐inflammatory cytokines (IL‐10 and TGF‐β) and antioxidant capacity (SOD) were decreased (Figure 7A). PL intervention successfully reversed the expression trends of these markers. Regarding mitochondrial function, mitoROS analysis confirmed the excessive accumulation of mitoROS in PIH placentas (Figure 7B), accompanied by a significant collapse of the mitochondrial membrane potential, as indicated by JC‐1 staining (Figure 7C). Furthermore, transmission electron microscopy revealed severe ultrastructural damage in PIH cells, including disrupted nuclear membranes, abnormal chromatin condensation, mitochondrial swelling, and cristae rupture. Remarkably, these functional and ultrastructural aberrations were substantially rescued in the PL intervention group (Figure 7D).

## 4 DISCUSSION

In recent years, the association between gut microbiota and maternal‐fetal health has garnered widespread attention. Emerging evidence suggests that maternal gut dysbiosis can promote hypertension and systemic inflammatory responses.^5^, ^21^ However, the precise functional niche of specific bacterial taxa and the causal relationship between their metabolites and the placental microenvironment remain elusive. SCFAs, as paramount metabolites derived from microbial fermentation, modulate host immune homeostasis, energy metabolism, and endothelial function^10^, ^11^, ^12^, ^22^; thus, their critical roles in obstetric complications are gaining increasing recognition. Our findings indicate that the beneficial effects of PL are likely dependent on the orchestrated regulation of SCFAs, including acetate, propionate, and butyrate. Notably, butyrate, as a key functional product, exhibits remarkable efficacy in promoting trophoblast invasion, alleviating OS, and dampening inflammatory cascades. By integrating single‐cell transcriptomics with in vivo functional assays, this study systematically delineates the core pathological features of the PIH placenta across multiple dimensions, encompassing cellular composition, intercellular cross‐talk networks, OS and pyroptosis pathways, and macrophage subpopulation dynamics and their transcriptional regulation. Furthermore, we provide compelling evidence that PL intervention exerts a profound protective effect in mitigating PIH‐associated placental injury.

Previous clinical cohorts have reported a marked depletion of SCFA‐producing commensals in preeclamptic women, which strongly correlates with elevated blood pressure and systemic inflammation.^23^ However, establishing a causal relationship between specific bacterial taxa and placental microenvironmental homeostasis has remained challenging. PL, a specific member of the *Prevotella* genus, has recently been implicated in alleviating cardiovascular immune toxicity and metabolic stress.^6^, ^7^ Building upon these findings, our study provides the first in vivo evidence that PL recolonization effectively halts PIH progression. We postulate that the therapeutic efficacy of PL is not derived from the direct colonization of the placenta but rather mediated through its robust capacity to ferment dietary fibers into SCFAs, establishing a remote “gut‐metabolite‐placenta” communication axis.

SCFAs, primarily acetate, propionate, and butyrate, are indispensable signaling molecules that regulate host immune tolerance and endothelial function.^24^ Consistent with reports indicating that maternal SCFA supplementation can prevent gestational hypertension,^11^, ^12^ our metabolomic profiling revealed a severe depletion of SCFAs in the PIH model, which was significantly reversed by PL administration. Biologically, SCFAs (especially butyrate) are known to bind to G protein‐coupled receptors (e.g., GPR41/43) or act as histone deacetylase inhibitors to exert potent anti‐inflammatory effects.^25^, ^26^ Our findings extend this paradigm to the obstetric field, suggesting that the restoration of the SCFA pool by PL is the pivotal upstream event that mitigates OS and promotes trophoblast invasion (as evidenced by recovered MMP2/9 expression) in the ischemic placenta.

A deeper mechanistic exploration in our study centered on the NLRP3 inflammasome and subsequent pyroptosis. Aberrant activation of NLRP3 triggers caspase‐1 cleavage and GSDMD‐dependent pore formation, which has been established as a major driver of trophoblast dysfunction and sterile inflammation in PIH.^16^, ^17^, ^18^ Although previous studies have predominantly evaluated NLRP3 activation in isolated cell types,^20^, ^27^ our scRNA‐seq data unveiled the multicellular complexity of this process. We discovered that OS and pyroptotic signatures are highly heterogeneous across the placental niche. Notably, prior research has shown that gut‐derived butyrate can inhibit NLRP3 activation in intestinal macrophages.^14^ Our data suggest a homologous mechanism in the placenta: PL‐derived SCFAs likely enter the maternal circulation to remotely dampen NLRP3‐mediated pyroptosis in the placental microenvironment, thereby preserving the structural integrity of the placental barrier and preventing the leakage of antiangiogenic factors into the maternal bloodstream.

The application of high‐resolution scRNA‐seq in our study unmasked immense cellular heterogeneity that bulk tissue analyses often obscure. Our scRNA‐seq data pinpointed that the OS and pyroptotic responses in the PIH placenta are highly cell‐type specific, with endothelial cells driving transcriptional reprogramming and macrophages dictating the inflammatory tone. A major novelty of our work is the precise identification of HBCs, fetal‐origin resident macrophages crucial for placental angiogenesis and immune tolerance,^28^ as central responders to PIH and PL intervention. Our trajectory and pySCENIC regulatory network analyses revealed that PIH severely depletes the HBC subpopulation and disrupts core TF modules, such as Nfkbia (an essential inhibitor of NF‐κB signaling^29^, ^30^) and Aif1. PL intervention specifically rescued this myeloid subpopulation and restored the Nfkbia‐associated regulatory network, fundamentally shifting the placental immune tone from a proinflammatory (M1‐like) to a tissue‐repairing (M2‐like) state. Furthermore, cell–cell communication analysis highlighted that PL repairs the defective cross talk between endothelial cells and SMCs, indicating that this microecological intervention functionally rehabilitates the intrinsic immune‐vascular network rather than merely acting as a systemic vasodilator.

Despite these comprehensive insights, several limitations warrant acknowledgment. First, our conclusions are derived from a chemically induced (L‐NAME) rodent model of PIH, which may not fully recapitulate the spontaneous pathophysiology of human preeclampsia. Thus, the clinical efficacy of PL and the exact threshold of SCFA alterations require validation in large‐scale prospective human cohorts. Second, although we mapped the SCFA‐NLRP3‐pyroptosis axis, the exact molecular sensors on placental macrophages (e.g., GPR43 dependency) mediating SCFA signals remain to be definitively proven using receptor‐knockout models. Future investigations addressing these specific molecular checkpoints will be instrumental in translating this microbiota‐targeted strategy into clinical obstetric practice.

## 5 CONCLUSION

By integrating single‐cell transcriptomic profiling with in vivo functional validation, this study systematically elucidates the key pathological hallmarks of the PIH placenta, encompassing cellular composition remodeling, aberrant intercellular communication networks, mitochondrial OS, NLRP3‐mediated pyroptosis, and macrophage transcriptional dysregulation. Furthermore, we provide the first evidence that PL intervention effectively ameliorates these pathological abnormalities through a multidimensional, orchestrated regulatory mechanism. Our findings not only offer novel mechanistic insights into the pathogenesis of PIH but also highlight that microbiota‐targeted microecological interventions, specifically leveraging the gut microbiota and their functional metabolites, hold great promise as innovative preventive and therapeutic strategies for hypertensive disorders of pregnancy.

## AUTHOR CONTRIBUTIONS

**Jingjing Wu**: Conceptualization; funding acquisition; supervision; writing—original draft; writing—review and editing. **Linlin Hu**: Investigation; methodology; data curation. **Peipei Chen**: Formal analysis; software; visualization. **Lihong Cai**: Validation; writing—review and editing. All authors have read and agreed to the published version of the manuscript.

## CONFLICT OF INTEREST STATEMENT

The authors declare no conflicts of interest.

## ETHICS STATEMENT

All animal experimental protocols were approved by the Experimental Animal Ethics Committee of Fujian Medical University (Approval No.: IACUC FJMU 2023‐Y‐1224) and were conducted in accordance with institutional and national guidelines for animal welfare and the ARRIVE guidelines for reporting animal research.

## PATIENT CONSENT

Not applicable.

## PERMISSION TO REPRODUCE MATERIAL FROM OTHER SOURCES

Not applicable.

## CLINICAL TRIAL REGISTRATION

Not applicable.

## Supporting information

Supporting Information S1

Figure S1

## Data Availability

The raw single‐cell RNA sequencing data generated in this study have been deposited in the NCBI BioProject database under accession number PRJNA1495226 (https://dataview.ncbi.nlm.nih.gov/object/PRJNA1495226). All other data supporting the findings of this study are available from the corresponding author upon reasonable request.
