# Supplementary material for: Prevotella loescheii attenuates pregnancy‐induced hypertension by reshaping placental immunity and halting macrophage pyroptosis
Source: J Cell Commun Signal. 2026 Aug 17;20(3):e70099. doi: 10.1002/ccs3.70099 (PMC13481707; doi:10.1002/ccs3.70099)
Supplement: Supplementary file 1 [file CCS3-20-e70099-s002.docx]

Supplementary Figure 1. KEGG pathway enrichment analysis of differentially expressed genes (DEGs) in myeloid cells
